# Nanopore-based detection and characterization of yam viruses

**DOI:** 10.1038/s41598-018-36042-7

**Published:** 2018-12-14

**Authors:** Denis Filloux, Emmanuel Fernandez, Etienne Loire, Lisa Claude, Serge Galzi, Thierry Candresse, Stephan Winter, M. L. Jeeva, T. Makeshkumar, Darren P. Martin, Philippe Roumagnac

**Affiliations:** 1 CIRAD, BGPI, Montpellier, France; 2 BGPI, INRA, CIRAD, SupAgro, Univ Montpellier, Montpellier, France; 3 CIRAD, UMR ASTRE, F-34398 Montpellier, France; 4 ASTRE, Univ Montpellier, CIRAD, INRA, Montpellier, France; 5 UMR 1332 BFP, INRA, University Bordeaux, CS20032, 33882 Villenave d’Ornon cedex, France; 6 DSMZ Plant Virus Department, Messeweg 11/12, 38102 Braunschweig, Germany; 7 ICAR-Central Tuber Crops Research Institute, Thiruvananthapuram, Kerala India; 8 Computational Biology Group, Department of Integrative Biomedical Sciences, Institute of Infectious Diseases and Molecular Medicine, University of Cape Town, Observatory, Cape Town 7925 South Africa

## Abstract

We here assessed the capability of the MinION sequencing approach to detect and characterize viruses infecting a water yam plant. This sequencing platform consistently revealed the presence of several plant virus species, including *Dioscorea bacilliform virus*, *Yam mild mosaic virus* and *Yam chlorotic necrosis virus*. A potentially novel ampelovirus was also detected by a complimentary Illumina sequencing approach. The full-length genome sequence of yam chlorotic necrosis virus was determined using Sanger sequencing, which enabled determination of the coverage and sequencing accuracy of the MinION technology. Whereas the total mean sequencing error rate of yam chlorotic necrosis virus-related MinION reads was 11.25%, we show that the consensus sequence obtained either by *de novo* assembly or after mapping the MinION reads on the virus genomic sequence was >99.8% identical with the Sanger-derived reference sequence. From the perspective of potential plant disease diagnostic applications of MinION sequencing, these degrees of sequencing accuracy demonstrate that the MinION approach can be used to both reliably detect and accurately sequence nearly full-length positive-sense single-strand polyadenylated RNA plant virus genomes.

## Introduction

Metagenomic approaches have enabled the discovery of hundreds previously unknown virus species^1–4^. These discoveries have strengthened our understanding of the ecological roles and impacts of viral communities, indicating that viruses are likely essential components of ecosystems as diverse as the human gut^5,6^ and the oceans^7,8^. For example, metagenomics approaches that consider the spatial arrangements of plant samples, and the precise environmental contexts of individual sampling sites have revealed the impact of agriculture on the distribution and prevalence of plant viruses at the ecosystem scale^9^. While the usefulness of viral metagenomics for diagnostics and viral surveillance is still debated^10–13^, these approaches have proven effective for the discovery of unknown etiological agents^12,14,15^. Specifically, in plant virology, viral metagenomics approaches focusing on the analysis of virus-derived small interfering RNAs (siRNA) has gained in popularity for the detection of both known and previously uncharacterized plant viruses within infected plants^2,16,17^.

Viral metagenomics methodologies, which until now have been primarily based on high-throughput second generation sequencing technologies (Roche 454, Illumina, Ion Torrent, etc.), have generated billions of ~150–300 nucleotides (nt) or of 21–24 nt small RNA (sRNA) reads and, depending on the sequencing coverage, have been successful for detecting and characterizing low-frequency genetic variants within viral populations^18^. Although viral metagenomics approaches have both expanded our view of global viral biodiversity, and paved the way towards a better understanding of viral ecology and evolution, several methodological biases undermine the effective analysis and taxonomic assignment of the sequence reads and contigs that have been generated using these approaches^11,19,20^.

One of the most important of these methodological issues relates to the production and analysis of short reads^21^. Whenever a known related reference sequence is unavailable to guide the mapping of short reads, the reads must be assembled *de novo* to obtain either full genome sequences, or large genomic sequence “contigs”. These *de novo* assembled sequences are in most cases likely to be chimaeras of reads from different viral variants^22,23^: a factor that should exclude these sequences from being used to make phylogenetic and demographic inferences. In addition, differentiation between exogenous and endogenous virus sequences remains difficult to achieve based on *de novo* assembled short read contigs because the genomic environment of these contigs is usually missing.

A second methodological issue with metagenomic approaches that employ second generation sequencing methods relates to the difficulty of creating scaffolds by chaining reads and contigs together. Failure to build large enough scaffolds or contigs can reduce the proportions of reads that can be reliably identified as being related to known virus species using alignment-based approaches such as BLAST^24^. This technical problem is compounded by the dearth of viral taxa that are represented in public nucleotide sequence databases such as GenBank. A consequence of this is that, up to 70% or more of the reads that are generated by some environmental viral metagenomic studies, end up being labeled as “dark matter” because they have no detectable homology to sequences within the public nucleotide sequence databases^19,25^.

Third generation sequencing techniques that are capable of generating much longer reads from individual RNA or DNA molecules promise to eliminate the need to assemble contigs *de novo* from short sequence reads. These techniques could therefore avoid the problems mentioned above^26^. Among the third-generation sequencing techniques is that implemented in the Oxford Nanopore MinION. The MinION has recently proved effective for delivering, in real time, average reads lengths of >15 kilobases^27–29^. While the per nucleotide error rate of the MinION long reads is frequently in excess of 20% – substantially higher than that of the shorter Illumina reads^30^ – the high degrees of genome coverage that are achievable together with post-sequencing data analysis can still yield consensus sequences that are >99% accurate^31^ and can be effective for identifying recombinant viral variants^32^. The MinION has been used to detect and determine the full (or nearly full) genome sequences of a range of animal and human viruses, including ebola^33^, dengue^34^, zika^35^, influenza^31^, cowpox^36^, and Ross River virus^37^. The only published applications of the MinION to sequencing plant virus genomes involved the detection of maize streak virus, maize yellow mosaic virus and maize totivirus in maize plants^38^ and plum pox virus in plum plants^39^.

Here, we aimed at using the MinION for detecting and characterizing several plant viruses infecting a single yam plant. This sequencing platform proved efficient for detecting Dioscorea bacilliform virus (DBV, *Badnavirus* genus), yam mild mosaic virus (YMMV, *Potyvirus* genus) and yam chlorotic necrosis virus (YCNV, *Macluravirus* genus). The full-length genome sequence of YCNV was determined using a Sanger sequencing approach to enable the comparative quantification of the degrees of sequencing coverage and accuracy using the Illumina and MinION platforms. We show that consensus sequences of YCNV whether obtained by *de novo* assembly or by mapping of the MinION reads to a reference genome were >99.8% identical to the Sanger-derived sequence of this virus, which indicates that the MinION should in a near future reliably enhance research on, and the monitoring of, plant viruses.

## Materials and Methods

### Plant material

A water yam plant (*Dioscorea alata*) infected by YMMV and YCNV was collected in Kerala (India), in 2010 and maintained *in vivo* at the DSMZ Plant Virus Department (PV-1066).

### Small RNA extraction and Illumina sequencing

Small RNA (sRNA) molecules, including small 21–24 nucleotides (nt) interfering RNAs, were extracted from symptomatic leaves of the water yam sample using a RNAzolRT kit (WAK Chemie, Steinbach, Germany) following the manufacturers protocol (RNAzol®RT Brochure, 2010, Molecular Research Center, Inc. Cincinnati, OH). The small RNA fraction was quantified in a Qubit fluorimeter using a Qubit RNA HS Assay Kit (Thermo Fischer Scientific, Waltham, USA) and checked for quality in a bioanalyser 2100 (Agilent). RNA passing the quality check was used for library preparation and subjected to high-throughput sequencing on an Illumina “Hi-Seq 2000” instrument using the services of a commercial company (Fasteris SA, Plan-les-Ouates, Switzerland).

### *In silico* screening of publically available expressed sequence tag (EST) data from yam

A systematic search of viral sequences in *de novo* assembled sequences (using the CAP3 sequence assembly program^40^) from publicly available *D*. *alata* expressed sequence tag (EST) resources (GenBank accession numbers: HO809681-HO825421, HO825422-HO840419 and HO850622-HO864016) was performed using BLASTn and BLASTx searches against the GenBank nucleotide collection (nt/nr) sequence databases implemented in the software KoriBlast 3.1 (KoriLog, Muzillac, France) with a maximum e-value threshold of 10^−3^.

### Characterization of the full-length genome sequence of yam chlorotic necrosis virus using Sanger sequencing

Total RNA from the water yam sample was extracted using the Qiagen® RNeasy Plant Mini Kit (Qiagen, Valencia, CA) as described by the manufacturer. A set of 30 overlapping primers (Supplementary Table 1) were designed based on an alignment (using MUSCLE^41^ with default parameters) of the consensus sequence of YCNV obtained using the sRNA Illumina approach, one 2306 nt long sequence with a high degree of similarity (80.7%) to YCNV recovered from *D*. *alata* EST sequences obtained from a plant grown in Nigeria^42^, and sequences of known macluravirus species (Chinese yam necrotic mosaic virus (CYNMV) and yam chlorotic necrotic mosaic virus (YCNMV)). RT-PCR reactions were performed using the Qiagen OneStep RT-PCR Kit (Qiagen, Valencia, CA). The 25 μL RT-PCR reaction mix consisted of 1 μL of eluted RNA, 14 μL of RNAse-free water, 5 μL of RT-PCR buffer (5X), 1 μL of dNTP mix (10 mM), 1.5 μL of each primer (10 μM) and 1 μL of RT-PCR enzyme mix. The RT-PCR program was as follows: 50 °C for 30 min, 95 °C for 15 min, 35 cycles at 94 °C for 1 min, 55 °C for 1 min and 72 °C for 1 or 2 min with a final 72 °C extension for 10 min. PCR products were analyzed by electrophoresis on a 1.2% agarose gel in TAE buffer stained with ethidium bromide and visualized under UV light. Since the extreme terminal ends were not covered, the exact termini were analyzed by 5′- and 3′-RACE. To amplify and sequence the 5′ region of YCNV genome, cDNAs were produced with the virus specific primer YamMac31R (Supplementary Table 1) using SuperScript III Reverse Transcriptase (Thermo Fischer Scientific, Waltham, USA). These cDNAs were subsequently tailed in parallel with polyA, G or C using a recombinant terminal transferase TdT enzyme mini kit (New England Biolabs, Ipswich, USA). The 5′RACE-PCR was performed as described in Knierim *et al*.^43^. The sequence of the 3′ end was determined with a 3′RACE-PCR procedure (Supplementary Table 1). Amplicons generated with poly-T primer and virus specific primer YAMMAC4F (Supplementary Table 1) were directly sequenced using automated Sanger sequencing (Genewiz, South Plainfield, USA). Assembly and alignment of the nucleotide sequences and the identification of ORFs was performed as previously described^44^. The polyA tail was further removed from the YCNV reference sequence to improve the reliability of alignment.

### Nanopore MinION library preparation and sequencing

One-hundred mg of yam leaves were ground with a mortar and pestle in liquid nitrogen and 450 µL of RLC buffer (Qiagen) with 0.1% of ß-mercaptoethanol (Sigma Aldrich) were added in a 1.5 ml tube, which was then vortexed twice and kept for five minutes at room temperature. Total RNA was isolated using the RNeasy Plant Mini Kit (Qiagen). A concentration of 25 ng/μl of total RNA was quantified by the Qubit fluorometer (Life technologies). The MinION sequencing library was prepared using the SQK-PCS108 cDNA-PCR kit (Oxford Nanopore Technologies Ltd). Briefly, cDNA was first generated using RT and the strand-switching method (Supplementary Fig. 1) and was then amplified by PCR using primers supplied in the SQK-PCS108 cDNA-PCR kit. Adapters also supplied in the SQK-PCS108 cDNA-PCR kit were then ligated to the PCR product (Supplementary Fig. 1). The eluted library was loaded onto a R9.4 Flow Cell  (FLO-MIN106 R9.4). Sequencing was performed using the MinION Mk1B device (MIN-101B). One strand of the duplex was sequenced at a time, producing 1D reads. The flow cell was run for 48 h using the standard MinKNOW software (version 1.11.5) and reads generated were base-called in real-time using the cloud-based Metrichor service provided by Oxford Nanopore Technologies.

### Illumina and MinION reads analyses

Illumina and MinION reads and contigs were compared to sequences in the GenBank database using DIAMOND^45^ searches for MinION reads or BLASTn and BLASTx^24^ searches for Illumina reads with a maximum e-value threshold of 10^−3^. Individual macluravirus-related MinION reads were then pairwise aligned with the YCNV Sanger reference genome (hereafter called the reference sequence) using a kmer-based approach (k = 8). Specifically, for each read (hereafter called a query sequence) identified as having detectable homology to the reference sequence, the coordinates of all perfectly matching 8 nt long query sequence fragments were identified on the reference sequence. All perfectly matching 8 nt fragments that were within 20 nucleotides of at least one other perfectly matching 8 nt long fragment on either the query or reference sequences were flagged as being accurately mapped and each of the nucleotides within these fragments was identified as being accurately sequenced (i.e. all eight nucleotides were denoted as “matches”). When 8mer fragments of the query sequence that contained nucleotides that were not denoted as matches in this initial scan (i.e. those nucleotides that were not within any 8 mer fragment that was accurately mapped), were bounded by two accurately mapped 8 mer fragments, the portion of the reference sequence between the mapping coordinates of these accurately mapped fragments were pairwise aligned (using ClustalW^46^; with default settings) with the unmapped query sequence nucleotides. Matching nucleotide positions, mismatched nucleotide positions and the numbers and lengths of insertions and deletions in the query sequence fragment were then enumerated from these pairwise alignments. The 3′ and 5′ unmapped fragments of the query sequences were not used to enumerate numbers of matching nucleotides, mismatching nucleotides, insertions and deletions.

### *De novo* assembly of Illumina sRNA and MinION sequencing data

Illumina reads cleanups and corrections were performed using the CutAdapt version 1.8.1 program^47^ and *de novo* assemblies were generated using SPAdes 3.6.2 software^48^ and CAP3^40^. MinION reads *de novo* assembly was done using Canu v1.6^49^ with the suggested parameters for Nanopore R9 1D sequencing (-nanopore-raw overlapper = mhap utgReAlign = true) using reads >1 kb (minReadLength = 1000) and a genome size of 20 kb (genomeSize = 20 k).

### Illumina sRNA and MinION sequencing coverage and accuracy

Both sets of reads were independently and jointly mapped to the complete Sanger YCNV genome with Bowtie2 v2.2.9^50^ using the end-to-end and very sensitive option. All reads mapped by Bowtie2 were kept in the analysis. Resulting bam alignment files were then processed with samtools 1.3^51^ to produce mpileup files. Bases with a phred score lower than 13 were filtered out. The resulting mpileup files were then parsed with custom python and R scripts to extract positions and coverages (second and fourth column, respectively), while the similarity was computed using the fifth column of the file, which displayed the numbers of matches, mismatches and indels at each position in the original bam files. Similarity was reported as percentage of match over the coverage. Means of similarity across 100 nt long sliding windows (step size: one nucleotide), were used for clarity of display. Dataset and scripts are available in a github repository at the address: https://github.com/loire/roumagnac2018_figs. Consensus sequences of the YCNV genome were obtained from mapped sRNA Illumina reads, mapped MinION reads, and jointly mapped sRNA Illumina plus MinION reads. Pairwise identity analyses of the reference YCNV Sanger sequence and *de novo* Minion contigs, mapped sRNA Illumina reads-, mapped MinION reads-, and mapped sRNA Illumina/MinION reads- consensus sequences were carried out using the MUSCLE-based pairwise alignment option implemented in SDT v1.2^52^.

### Pairwise identity and phylogenetic analyses

All available macluravirus full genomes were downloaded from GenBank and aligned using MUSCLE^41^. Maximum-likelihood phylogenetic trees were inferred using PhyML 3.1^53^ implemented in MEGA version 6.06^54^ with the nucleotide substitution model GTR + G + I selected as best-fit models by jModelTest^55^. Branch supports were tested using 1000 bootstrap replicates.

## Results

### Taxonomic assignment of virus-related reads produced by the MinION sequencing technology

With the intention of comparing the potential utility of Illumina and MinION sequencing platforms within a virus discovery context, we attempted to determine the complement of viruses present within a single yam plant using a Nanopore MinION device. In total, 2,036,598 reads were generated of which 41,487 (2%) were identified as likely being virus-derived using DIAMOND searches (Table 1). While most of these viral reads were assigned to YMMV (22,055 reads, 53.2%) and YCNV (19,252 reads, 46,4%), 156 reads (0.4%, 1.2 × 10^−4^ < DIAMOND e-values < 1.7 × 10^−34^) were related to DBV, 17 to pestiviruses (0.04%, 7.5 × 10^−6^ < DIAMOND e-values < 7.4 × 10^−15^), 5 to bymoviruses (0.01%, 3.8 × 10^−19^ < DIAMOND e-values < 3.4 × 10^−37^) and 2 to begomoviruses (0.005%, 8 × 10^−4^ < DIAMOND e-values < 1.2 × 10^−10^). Interestingly, DBV is reportedly the most prevalent virus in yam^56^ and transcriptionally active endogenous geminiviral (EGV) sequences have also been identified integrated within the genomes of many yam species^57^. The two begomovirus MinION reads recovered in this study share 67% and 89% identities with previously identified EGVs in *D. alata*. Five reads were assigned to the *Bymovirus* genus: a sister genus of the *Macluravirus* genus in the *Potyviridae* family. These reads may have been misassigned due to the potentially high rate of MinION sequencing errors and may actually be macluravirus-related reads.

**Table 1:** Viral detection using total RNA extraction and the Nanopore MinION sequencing technology.

Taxonomic assignment (DIAMOND)	Number of reads	Percentage of total viral reads	Minimum read length (bp)	Maximum read length (bp)	Average read length (bp)
Badnavirus	156	0.4	354	3112	1420
Begomovirus	2	5.10^−3^	330	1560	945
Bymovirus	5	1.10^−2^	331	2460	1510
Macluravirus	19252	46.4	236	14211	1104
Pestivirus	17	4.10^−2^	356	1071	613
Potyvirus	22055	53.2	227	8876	1163

### *De novo* assembly of Illumina and MinION reads

A total of 15,365,074 small RNA - including small interfering RNA - raw Illumina sequence reads were generated from the water yam sample. In agreement with the analysis of the MinION reads, 56 contigs >100 nt long that were obtained by *de novo* assembly of Illumina reads showed significant degrees of similarity to badnaviruses, macluraviruses and potyviruses based on BLASTx searches (Table 2). Overall, these contigs were shorter (maximum length of 2424 nt) than the MiniION reads. While contigs sharing identities to begomoviruses, bymoviruses and pestiviruses were not recovered using *de novo* assembly of Illumina reads, 4 contigs related to ampeloviruses were identified (5.0 × 10^−5^ < BLASTx e-values <3.0 × 10^−14^).

**Table 2:** Viral detection using sRNA extraction and the Illumina sequencing technology.

Taxonomic assignment (BLASTx)	Number of contigs (>100 bp)	Contig lengths (bp)	Average contig lengths (bp)
Ampelovirus	4	170–201	189
Badnavirus	4	155–524	342
Macluravirus	18	147–2118	543
Potyvirus	30	102–2424	458

*De novo* assembly of MinION reads yielded two large contigs. BLASTn comparisons between these two contigs and all sequences in GenBank indicated that the highest similarity scores were detected with YMMV (accession number KJ125479, highest percent nucleotide identity = 85%, query coverage = 99%, e-value = 0) for contig #1 (8791 bp) and YCNV (accession number MG755240, highest percent nucleotide identity = 82%, query coverage = 99%, e-value = 0) for contig #2 (7277 bp).

### Characterization of the full-length genome sequence of yam chlorotic necrosis virus

**Figure 1:**
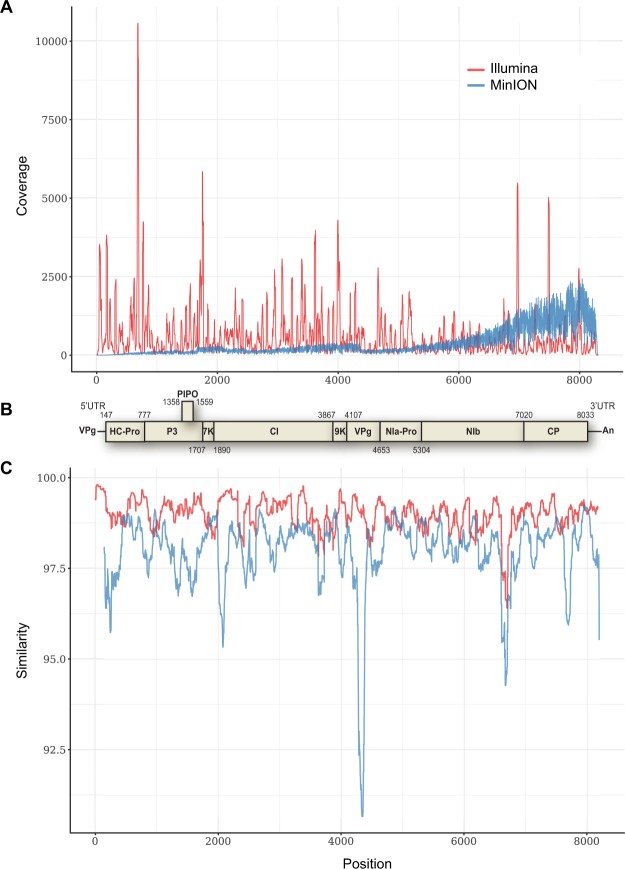
Comparison of the (**A**) coverage and (**C**) similarity of MinION and Illumina reads when aligned to the reference yam chlorotic necrosis virus (YCNV) genome sequence. (**B**) Genome organization of YCNV. The ORFs that are likely to represent genes expressing characteristic macluravirus proteins were identified based on comparisons with other members of the genus *Macluravirus*, including the helper component-proteinase (HC-Pro), P3, 7 K, the cylindrical inclusion putative helicase (CI), 9 K, the genome-linked protein (VPg), the nuclear inclusion protease (NIa-Pro), the nuclear inclusion polymerase (NIb) and the coat protein (CP).

A 8263-nt full length genome sequence of YCNV isolate Kerala (accession number MH341583) was further assembled and annotated, confirming that it possesses a typical macluravirus genome organization (Fig. 1B), including a large open reading frame (ORF) predicted to translate into a 2628 aa long polyprotein (Fig. 1B). This large unique ORF, which starts at position 147 and ends at position 8033, was predicted by analogy with other macluraviruses to encode nine functional products after cleavage, including a helper component-proteinase (HC-Pro), P3 (protein of unknown function), 7 K (protein of unknown function), a cylindrical inclusion putative helicase (CI), 9 K (protein of unknown function), a genome-linked protein (VPg), a nuclear inclusion protease (NIa-Pro), a nuclear inclusion polymerase (NIb) and a coat protein (CP)^58,59^. Like the other known annotated macluravirus genomes, this virus lacks a P1 proteinase, and is likely to use its HC-Pro and NIa proteinases for polyprotein cleavage. The HC-Pro self-cleavage site between HC-Pro and P3 is likely the FVG/V site located at aa positions 259 to 262 of the polyprotein.

Based on the alignment of the 2628 aa long polyprotein with artichoke latent virus (ArLV) Chinese yam necrotic mosaic virus (CYNMV) and YCNV isolate YCNV-YJish from China^60^, five putative NIa proteinase cleavage sites were identified that have the canonical dipeptide Q(E)/M(A) with a conserved L residue at the −2 position from the cleavage site. These potential cleavage sites were located at positions 569 (Q/A, cleaves between P3 and 7 K), 630 (Q/M, 7K-CI), 1289 (Q/A, CI-9K), 1369 (E/M, 9K-NIa), and 2340 (E/M, NIb-CP) of the polyprotein (Fig. 1B). Two additional putative cleavage sites containing noncanonical dipeptides, including E/I (1551, VPg-NIa) and Q/H (1768, NIa-NIb) were also identified. Cleavage of the polyprotein therefore yields mature proteins of the following sizes: HC-Pro (261 aa), P3 (309 aa), 7 K (62 aa), CI (660 aa), 9 K (81 aa), VPg-NIa (400 aa, and 183 for VPg alone), NIb (573 aa) and CP (289 aa). As identified in other macluraviruses, a P3N-PIPO ORF that is classically found in *Potyviridae* members^61^ was identified within the P3 ORF. The consensus polymerase slippage GAAAAAA sequence (nt positions 1358 to 1364) is present, followed by a short PIPO region of 59 aa, ending at a stop codon at positions 1538–1540 of the genome.

**Figure 2:**
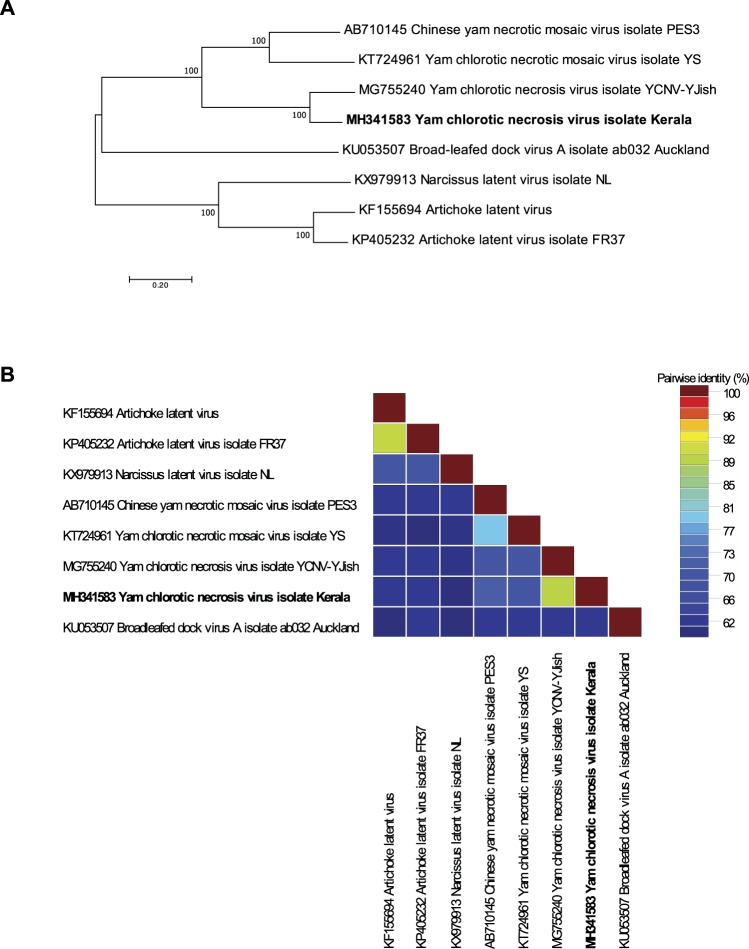
(**A**) Maximum likelihood phylogenetic tree of complete macluravirus genome nucleotide sequences. Branch supports were tested using 1000 bootstrap replicates. (**B**) Pairwise genome-wide sequence identities of the macluravirus genome nucleotide sequences.

A phylogenetic tree based on whole genome sequences of known macluraviruses indicated that YCNV isolate Kerala clusters with the other described macluraviruses (Fig. 2A), its closest relatives being YCNV isolate YJish from China and two other macluraviruses isolated from yam: CYNMV and yam chlorotic necrotic mosaic virus (YCNMV)^59,62^. The virus sharing the highest genome-wide pairwise identity with YCNV isolate Kerala (81.9%) is YCNV isolate YJish (Fig. 2B).

### Total error rate assessment of YCNV-related MinION reads

The total mean sequencing error rate of the 19,252 YCNV-related MinION reads was 11.25%, including substitutions, insertions and deletions. In addition, the average numbers of insertions per nucleotide per read and the average number of deletions per nucleotide per read were 0.003% and 0.015%, respectively. Noteworthy, the alignment-based approach that was used for the calculation of the total mean sequencing error rate revealed that several MinION reads were composed of non-contiguous regions of the YCNV genome, suggesting that either defective viral variants were sequenced or that the MinION generated chimeric sequences.

### Genomic coverage and genome sequencing depth of YCNV sequences obtained using *de novo* assembly or mapping of Nanopore and Illumina reads

*De novo* assembly of the MinION reads, yielded a large contig (contig #2) that covered 87.5% of the full-length YCNV genome and shared 99.889% pairwise identity with the reference Sanger sequence of this genome (Table 3). The 5′ part of the YCNV sequence was missing from this MinION contig. *De novo* assembly of the Illumina reads yielded 18 contigs that were further chained together to form a single scaffold that covered 88.1% of the full-length YCNV genome and shared 99.739% pairwise identity with the reference Sanger sequence of this genome (Table 3).

**Table 3:** Coverage and identity of consensus mapped and *de novo* assembled genome sequences of yam chlorotic necrosis virus (YCNV) generated from Illumina and MinION reads.

YCNV consensus sequence	Length (bp)	Coverage (%)	Identity (%)
*De novo* assembly of sRNA Illumina reads and scaffolding of the contigs	7280	88.1	99.739
*De novo* assembly of MinION reads	7231	87.5	99.889
Mapping of MinION reads against YCNV Sanger genome sequence	7970	96.5	99.837
Mapping of sRNA Illumina reads against YCNV Sanger genome sequence	8263	100	99.891
Mapping of MinION and sRNA Illumina reads against YCNV Sanger genome sequence	8263	100	99.927

**Figure 3:**
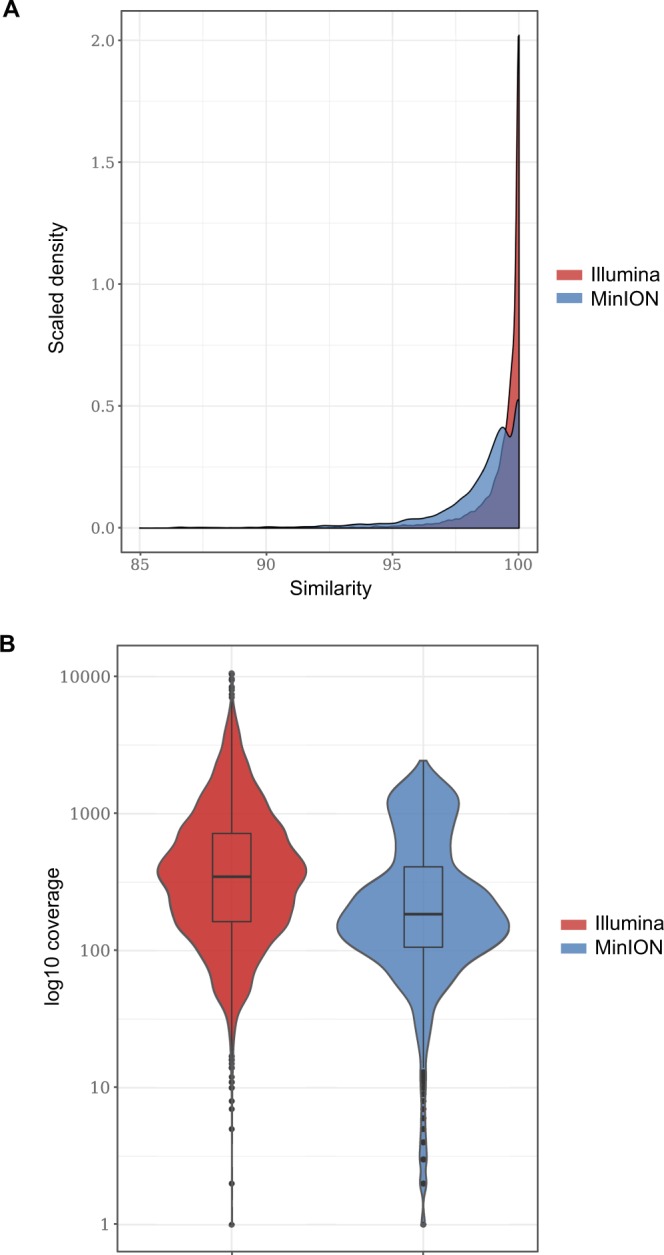
(**A**) Densities of the similarity values of the Illumina and MinION reads that were mapped to the reference YCNV genome. (**B**) Degree of YCNV genome sequencing depth for the Illumina and MinION technologies.

A total of 5713 and 293,986 of the MinION and Illumina reads were further mapped to the complete YCNV Sanger genome, respectively. The similarity of the Illumina reads mapped to the reference YCNV genome was higher than the similarity of the mapped MinION reads (Fig. 3A). In addition, the degree of genome sequencing depth was higher for the Illumina technology (615-fold for Illumina vs. 366-fold for MinION, Fig. 3B). The distribution of the Illumina and MinION reads mapped to the reference YCNV genome also differed. While the Illumina reads were quite evenly distributed on the reference sequence with some hot spots of deeper coverage, the MinION reads were predominantly distributed towards the 3′ part of the reference genome (Fig. 1A). In both the MinION and Illumina derived macluravirus genome sequences, sequencing errors were quite evenly distributed across the YCNV genome (Fig. 1C). Mapping of the MinION reads against the Sanger YCNV reference sequence yielded a consensus YCNV sequence that covered 96.5% of the reference genome and shared 99.837% pairwise identity with it (Table 3). On the other hand, mapping of the Illumina reads against the YCNV Sanger reference yielded a consensus that covered the entire YCNV sequence and shared 99.891% pairwise identity with it (Table 3). Finally, mapping both MinION and Illumina reads together against the YCNV reference yielded a consensus sequence covering the entire YCNV genome and sharing 99.927% pairwise identity with it (Table 3).

## Discussion

While the MinION sequencing approach has recently proved to be a reliable real-time portable technology that enhances research and monitoring of human and animal viruses^31,33–37^, to date this technology has seldom been used in plant virology^38,39^. Here we assessed the capability of this sequencing approach to detect and characterize viruses infecting a water yam plant. While our overarching goal was to assess whether the MinION should be used in plant virology to enhance research on, and the monitoring of, plant viruses, we challenged the MinION technology with the well-established Illumina sequencing technology and compared the results obtained using these two sequencing approaches.

Three viruses, including a badnavirus, a macluravirus, and a potyvirus were detected using both approaches. Other viral sequences were revealed by only one or the other of the two sequencing approaches, including pestivirus- and begomovirus-related sequences revealed by the MinION and ampelovirus-related sequences revealed by Illumina. To date, the *Pestivirus* genus is not known to contain any plant virus species. Hence, it is plausible that the occurrence of pestivirus-related reads is a consequence of contamination by genetic material from an animal virus. The two begomovirus-related MinION reads may indicate the presence of transcribed endogenous geminiviral sequences^57^. This is because the MinION protocol developed in this study specifically targeted poly(A) sequences: which would have presumably been plant or plant-associated bacterial/fungal derived polyadenylated mRNA transcripts (Supplementary Fig. 1). Interestingly, it is possible that the analysed yam sample was infected by a novel ampelovirus that went undetected by the MinION approach. Specifically, ampeloviruses have positive sense ssRNA genomes lacking a 3′ poly(A) tract and were probably not amplified using the MinION amplification strategy used in this study (Supplementary Fig. 1) and the transcripts of this non-polyadenylated virus were maybe far too few to be detected by the MinION.

Overall, the present study shows that the MinION is an efficient means of detecting and characterizing positive sense ssRNA viral genomes that have 3′ poly(A) tracts. It also indicates that the *de novo* assembly of MinION reads can yield nearly complete high-quality genome sequences for plant virus species. However, the study also reveals that plant viruses lacking a 3′ poly(A) tract (e.g. ampeloviruses) could potentially go undetected using the MinION protocol that was applied here. This bias would probably not have been an issue if viral genomes were randomly amplified. Interestingly, a recent study has shown that MinION enables rapid detection of enteric viruses following the ligation of sequencing adapters to randomly pre-amplified viral genomes^63^. Specifically, viral metagenomics approaches based on random amplification of virion-associated nucleic acids (VANA) purified from virus-like particles have proven effective in the discovery of novel RNA and DNA plant viruses^2,64,65^, which paves the way towards sequencing all types of plant viruses using the MinION technology. In addition, MinION 1D^2^ libraries should be used because they offer increased accuracy advantages relative to the existing 1D approach. However, PCR-free MinION detection of plant viruses may be problematic because of how technically difficult and time-consuming it is to purify large-enough quantities of plant virus DNA/RNA from plant tissue samples.

Sanger sequencing of the yam-infecting macluravirus provided a standard against which we could compare the degree of genome coverage and sequencing accuracy achieved by the MinION and Illumina approaches. However, before using the genome of this macluravirus as a guiding sequence, we tentatively taxonomically assigned it. The virus sharing the highest genome-wide pairwise identity with YCNV isolate Kerala (81.9%) is YCNV isolate YCNV-YJish (Fig. 2B). This degree of similarity is above the species demarcation thresholds recommended for all of the different *Potyviridae* genera (nt sequence identity less than 76% either in the coat protein gene or over the whole genome), suggesting that the water yam plant used in this study is infected by YCNV. While the genome coverage of the mapped Illumina reads was better than that of the mapped MinION reads, the consensus YCNV sequences generated by both approaches shared more than 99.83% identity with the YCNV reference Sanger sequence (Table 3). Combining both the MinION and Illumina reads yielded a consensus YCNV sequence sharing 99.93% identity with the Sanger determined YCNV sequence (Table 3). This degree of accuracy translates to a maximum of six errors over a 8263 nt genome. Considering the known intra-isolate variability of RNA viruses, and that sequences were determined from different plant parts collected at different times for the MinION, Illumina and Sanger sequencing approaches, this “maximum error rate” is remarkably low as some of the identified differences likely reflect actual differences between the sequenced viral populations and not sequencing errors.

Besides this promise, the potential utility of the MinION platform for generating sequencing reads in excess of 10 Kb could have a major impact on viral metagenomics studies. Reads of this length would encompass the genomes of most viruses with RNA or ssDNA genomes. This would correct two of the most important biases that arise during metagenomics studies^11,20^. First, long reads would solve the assembly chimaera problems that have plagued attempts to use short-read sequencing platforms such as Illumina for sequencing genomes directly from non-clonal viral populations. Secondly, long-read sequencing platforms like MinION will yield smaller numbers of unassigned reads (i.e. will reduce the amount of dark matter) by increasing the efficiency with which reads can be assembled into contigs. While the sizes of these contigs will be larger, the probability that highly variable genes (such as silencing suppressor or movement protein encoding genes) will be accurately linked within contigs to more conserved genes (such as coat proteins, polymerases or replication-associated genes) will be increased.

However, the per-read error rate of the MinION remains very high (most often exceeding 10%) and the total numbers of sequenced nucleotides are lower than Illumina and other shorter reads platforms. Also, it remains to be determined whether the MinION platform is capable of differentiating between exogenous and endogenous viral sequences, between the different components of segmented virus genomes or between viral variants. These limitations could hinder reliable *de novo* assembly of MinION reads, which might still result in chimeric consensus sequences that mix reads originating from different viral variants. Consequently, there are several areas in which improvement is still needed before sequences generated by this technology can be used reliably for downstream analyses such as to test for evidence of genetic recombination, adaptive evolution, or resistance breaking mutations.

Nevertheless, successfully demonstrating that the MinION approach can be used to both reliably detect and accurately sequence nearly full-length plant virus genomes is an important first step towards the application of highly portable sequencing platforms like the MinION to field-based plant virus diagnostics such as has already been attempted for the identification of closely-related plant species^66^ and the mobile real-time surveillance of Zika viruses in Brazil^67^. While field-monitoring of plant viral diseases is a still-missing first step towards identifying and controlling the emergence of new plant diseases, widespread use of highly portable sequencing platforms like the MinION is likely to revolutionize plant disease diagnostics and is already poised to enable the implementation of reliable epidemiosurveillance networks.

## Electronic supplementary material

Supplementary Material
