# Supplementary material for: Nanopore-based detection and characterization of yam viruses
Source: Sci Rep. 2018 Dec 14;8:17879. doi: 10.1038/s41598-018-36042-7 (PMC6294787; doi:10.1038/s41598-018-36042-7)

Nanopore-based detection and characterization of yam viruses

Denis Filloux, Emmanuel Fernandez, Etienne Loire, Lisa Claude, Serge Galzi, Thierry Candresse, Stephan Winter, M. L. Jeeva, T. Makeshkumar, Darren P. Martin, and Philippe Roumagnac

Supplementary Table 1. Primers used for sequencing (Sanger) yam chlorotic necrosis virus complete genome

Primers code	Primers	Sequences	Positions	Authors
P1	ANCHOR-DT-V	CACGCGTATCGATGTCGAC(T)16V	3' and A-tailed-5' end	Roche[(C)]
P2	ANCHOR	CACGCGTATCGATGTCGAC	3' and A-tailed-5' end	Roche[(C)]
P3	M4T	GTTTTCCCAGTCACGAC(T)15	3' and A-tailed-5' end	Chen *et al.*, 2001[1]
P4	M4	GTTTTCCCAGTCACGAC	3' and A-tailed-5' end	Chen *et al.*, 2001[1]
P5	poly-C	ATCCTGCAGGCGCG(C)16	G-tailed-5' end	Knierim *et al.*, 2015[2]
P6	poly-C-D	ATCCTGCAGGCGCG(C)16D	G-tailed-5' end	Palanga *et al.*, 2017[3]
P7	poly-G	CTCAAACAGTCAC(G)15	C-tailed-5' end	Knierim *et al.*, 2015[2]
P8	poly-G-H	CTCAAACAGTCAC(G)15H	C-tailed-5' end	Palanga *et al.*, 2017[3]

P9	YamMac54F	AAAATTAAACAATTGAATGCAACTA	1	This study
P10	YamMac30R	GTGTCTTACCGATTTTTGCGA	126	This study
P11	YamMac21F	ACTGSAACTCATGGCAACTG	137	This study
P12	YamMac22R	ACAGTTGCCATGAGTTSCAG	157	This study
P13	YamMac31R	GTTTTATCAGGAATTGTCAATG	256	This study
P14	YamMac32F	CGATGTAATGAGAGAACTTGG	671	This study
P15	YamMac23F	CACATCTGTGATCAGAGAGG	810	This study
P16	YamMac23R	CCTCTCTGATCACAGATGTG	829	This study
P17	YamMac33R	AGCTTCCCTTAATAAGACCTG	906	This study
P18	YamMac34F	GTGAAAAGAAGATGATGTCTGC	1870	This study
P19	YamMaclura18F	TGCGATGGCAATGATAACGC	1889	This study
P20	YamMaclura17R	ACCACTCCTGATGTTGTGTC	2014	This study
P21	YamMac35R	TATCCTGTCCAACTTGAATAGC	2259	This study
P22	YamMaclura15F	GAAAGAARGAATGYGATCGAG	2836	This study
P23	YamMaclura14R	GGTGTGTTSGTGAATCCGAA	3055	This study
P24	YamMaclura12F	ATGTGGGATGATGAWGAYGG	4176	This study
P25	YamMaclura11R	CATCATYGGRTTTCTTCTGTC	4298	This study
P26	YamMac24R	GCAGTTTTCATGGATCAYAAC	4502	This study
P27	YamMaclura10F	GTGATGGTATGATYGTGCAGA	5089	This study
P28	YamMaclura9R	GTTGGWATYTGATGAGCCCA	5206	This study

P29	YamMac36F	TTAAGAGAGCAGGAATGACTG	5863	This study
P30	YamMaclura7AR	CTTCTTCATGGCATACATTGG	5966	This study
P31	YamMac37F	CAGCAAAACACTTCGTTGATG	6157	This study
P32	YamMac38R	CCACTACCGTGTAGCCATC	6304	This study
P33	Sprimer	GGNAAYAAYAGYGGNCARCC	6489	Chen *et al.*, 2001
P34	YamMac39R	ATAGACCAATTCCAGTTCACC	6719	This study
P35	YAMMAC1F	ATYGARTGGMGRATHCCWGC	7335	This study
P36	YAMMAC2R	CCRTTYGCRYGDAYTTCCAT	7639	This study
P37	YAMMAC4F	CHGCAGCWATYGGKMGTG	7885	This study
P38	YAMMAC3R	ACKMCCRATWGCTGCDGC	7901	This study
P39	YAMMAC5R	GGGTTGCTGAGCRTYGGA	8178	This study

Legend: Summary of the different primers combinations

YCNV internal region sequencing

- PCR: P9/P10, P9/P12, P9/P13, P11/P16, P11/P20, P14/P17, P15/P20, P18/P21, P19/P23, P19/P25, P22/P25, P24/P26, P24/P28, P27/P30, P29/P32, P29/P34, P31/P32, P31/P34, P33/P36, P33/P38, P33/P39, P35/P39.

YCNV 5' region sequencing

- 5'RACE-PCR: P13 in RT, then P1/P12, P3/P12, P5/P12, P6/P12, P7/P12, P8/P12 in PCR followed by P2/P10, P4/P10, P5/P10, P6/P10, P8/P10 in nested-PCR.

YCNV 3' region sequencing

- 3'RACE-PCR: P37/P3-P4 in RT-PCR.

References

1 Chen, J., Chen, J. & Adams, M. J. A universal PCR primer to detect members of the Potyviridae and its use to examine the taxonomic status of several members of the family. *Arch. Virol.* **146**, 757-766, doi:DOI 10.1007/s007050170144 (2001).
2 Knierim, D., Maiss, E., Menzel, W., Winter, S. & Kenyon, L. Characterization of the complete genome of a novel Polerovirus infecting *Sauropus androgynus* in Thailand. *Journal of Phytopathology* **163**, 695-702, doi:10.1111/jph.12365 (2015).
3 Palanga, E. *et al.* Complete genome sequences of cowpea polerovirus 1 and cowpea polerovirus 2 infecting cowpea plants in Burkina Faso. *Arch Virol* **162**, 2149-2152, doi:10.1007/s00705-017-3327-z (2017).

Supplementary Figure 1. Protocol of cDNA production using RT-PCR and the strand-switching method and amplification by PCR using primers supplied in the SQK-PCS108 PCR-cDNA kit from Oxford Nanopore.

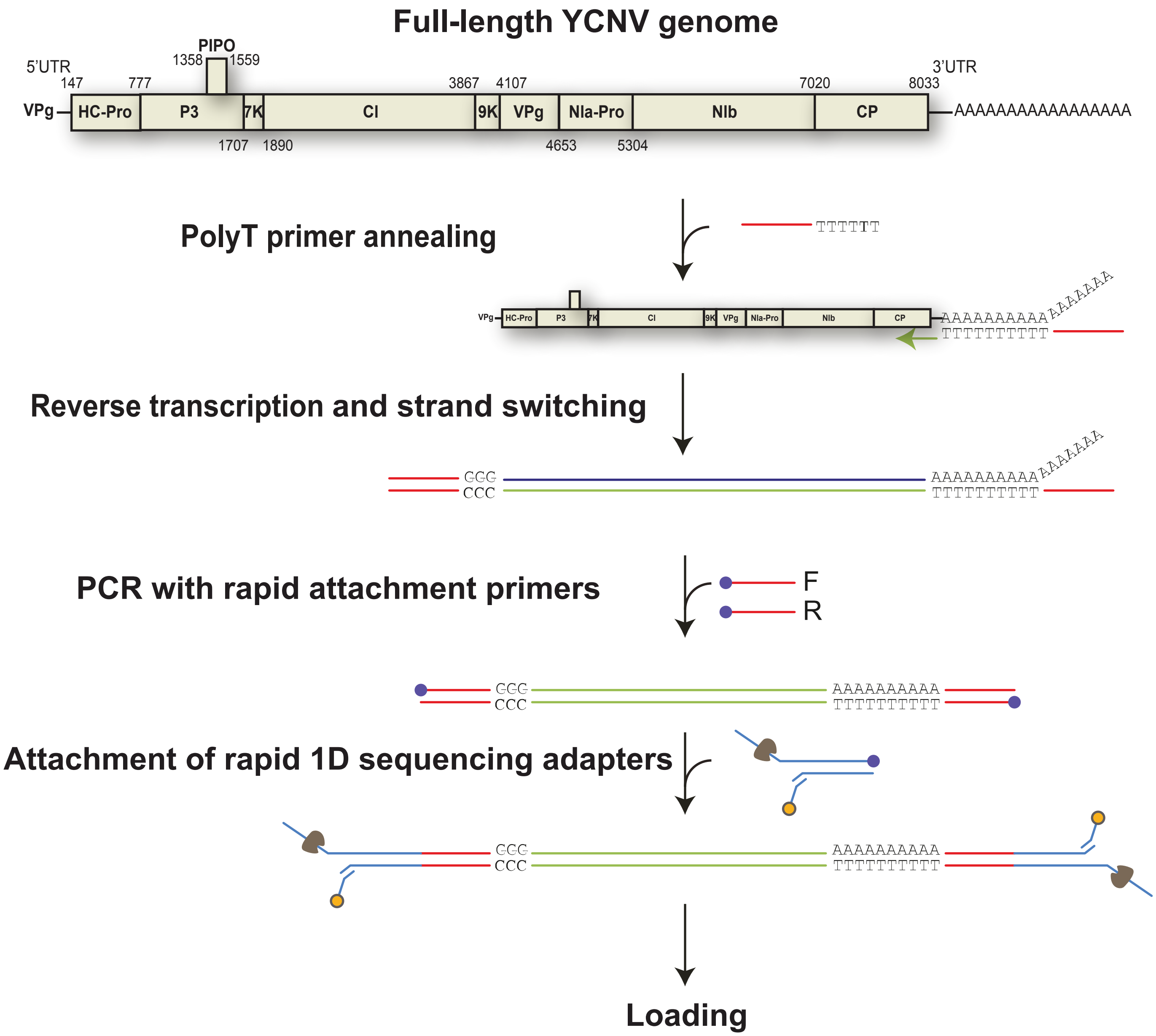

Supplement: Supplementary file 1 — Supplementary Material [file 41598_2018_36042_MOESM1_ESM.pdf]
